# Cognitive and Learning Outcomes in Late Preterm Infants at School Age: A Systematic Review

**DOI:** 10.3390/ijerph18010074

**Published:** 2020-12-24

**Authors:** Sílvia Martínez-Nadal, Laura Bosch

**Affiliations:** 1 Department of Pediatrics, SCIAS-Hospital de Barcelona, 08034 Barcelona, Spain; smartinez@sciashdb.com; 2 Institute of Neurosciences of the University of Barcelona (UBNeuro), 08035 Barcelona, Spain; 3 Institut de Recerca Sant Joan de Déu (IRSJD), 08950 Esplugues de Llobregat, Spain

**Keywords:** late preterm children, assessment tools, cognitive functioning, learning outcomes, executive function, language skills, academic achievement, school-age, neurodevelopment risk

## Abstract

Late preterm children born between 34^0/7^ and 36^6/7^ weeks’ gestation account for ≈70% of prematurely born infants. There is growing concern about this population at risk of mild neurodevelopmental problems, learning disabilities and lower academic performance. Following the Preferred Reporting Items for Systematic Review and Meta-Analysis (PRISMA) statement, this paper analyzes recent published evidence from 16selected studies involving late preterm children and control group assessments at preschool and/or school age, mainly focusing on cognitive functioning, language learning and academic achievement. The review identifies the assessment tools used in these studies (standardized tests, parental questionnaires and laboratory tasks) and the areas being evaluated from preschool (age 3 years) to primary school levels. Results reveal the presence of mild difficulties, pointing to suboptimal outcomes in areas such as executive function, short term verbal memory, literacy skills, attention and processing speed. Some difficulties are transient, but others persist, possibly compromising academic achievement, as suggested by the few studies reporting on higher risk for poor school performance. Given the increasing number of late preterm children in our society the review highlights the need to implement screening strategies to facilitate early risk detection and minimize the negative effects of this morbidity in childhood.

## 1. Introduction

Infants born between 34^0/7^ and 36^6/7^ weeks’ gestation have for a long time been considered low-risk preterm infants, also called near-term infants. They usually receive no special healthcare and are being treated as equivalent to infants born beyond 37 weeks of gestation in terms of their biomedical risk. It was not until the last decade that the label near-term was gradually replaced by late preterm, as suggested by the National Institute of Child Health and Child Development (NICHD) after a workshop celebrated in 2005 in which the need for specific research on the outcomes of this group of preterm babies was emphasized: clinical practice guidelines for this population should be developed and the assimilation of late preterm to term-born infants had to be reconsidered [1]. Since then, there has been growing concern about this population and, although short-term outcomes have begun to be analyzed [2,3,4], research on late preterm long-term outcomes is still rather limited. Studies in the last decade have reported mild developmental problems, learning disabilities, emotional and behavioral problems, and lower academic performance at school age [5,6,7,8,9,10,11,12]. Some research has also reported morbidity that may persist into adulthood [13,14,15,16,17] and, although the rate of long-term morbidities is not high, it is nevertheless greater than in term-born infants. The growing interest in this population arises from the fact that late preterm newborns represent the largest segment of the preterm neonatal population, accounting for approximately 70% of all prematurely born infants [18,19,20] so, even if it is just a percentage of them that will need some kind of developmental support or intervention, the total numbers are considerably great. The emotional and financial costs for late preterm children, their families and the health care system are additional factors that must certainly be taken into account.

Different studies have been carried out specifically exploring the nature of the structural and functional brain basis underlying late preterm outcomes, thus contributing to a better understanding of the behavioral findings obtained. These studies show that late preterm birth is generally associated to around 20% to 30% smaller brain volume, myelinization is less developed and gyral folding more immature than term-born. These differences had been related to developmental delays observed by age 2 years [21]. Alterations in brain white matter microstructure and connectivity at term-equivalent age relative to term-born children have also been observed [21,22,23,24,25,26]. Although brain imaging studies on late preterm are still scarce (and unfortunately some of the studies group together data from late and moderate preterm newborns), they converge in revealing the disruptive effect on brain development that preterm birth taking place in the later weeks of pregnancy might have, by somewhat altering the growth trajectories of cortical and subcortical structures that are key to neurodevelopment. Research still needs to be expanded in this domain to better predict the nature and scope of the neurodevelopmental risk in this population and to shed more light on the underlying connection between the neuroanatomical and functional findings obtained near or at term age and later neurobehavioral measures. The role of brain plasticity in response to early stimulation to compensate for this early vulnerability in late preterm birth deserves further attention. 

Considering that late preterm have been reported to be at risk for neurodevelopment outcomes, it is nevertheless surprising that they do not receive a specific developmental assessment in their early years, despite the recommendations of different scientific societies [27,28,29,30]. Critically, many of the studies found in the literature show methodological heterogeneity, a factor that constrains reaching solid or more nuanced conclusions about the real impact of late prematurity on neurodevelopment and (long-term) academic outcomes.

Our review strategy for selecting the studies diverges from the one adopted in previous systematic reviews published in the last decade [31,32,33]. McGowan et al.’s review [31], published in 2011, was centered on data from studies of late preterm found in publications spanning from 1980 to 2010, with a focus on early childhood (from 1 to 7 years of age), health, neurodevelopment and education skills, but including heterogeneous samples of both late preterm admitted for Neonatal Intensive Care and healthy non-admitted late preterm. Another systematic review [32] appeared in 2015 and was focused on the long-term outcomes of late preterm in the motor, cognitive, language and academic domains, reported in studies covering the wide age range 12 months to 18 years and published between 2000 and 2013. Similarly, Chan et al.’s review [33] covered studies published up to year 2013 on cognitive and educational outcomes from late preterm but also including comparisons with full term and early term children. The aim of the present review is to update the knowledge on late preterm learning outcomes and academic performance, focusing on evidence published in the last decade (2010–2019), from studies comparing healthy late preterm children and at term control groups, assessed at some point during pre-school, kindergarten and early school years, and corresponding to cohorts of participants born not earlier than the late 90s (studies based on cohorts born in the 80s or early 90s were excluded). We are selectively interested in studies reporting measurements of learning outcomes, cognitive functioning and/or language skills relevant to academic achievement. Although we were particularly interested in studies adopting a longitudinal approach (two follow up assessments on the same group), those reporting outcomes from a single age group are also included. We aim at identifying types of assessment tools and variables measured adequate to evaluate late preterm children outcomes at different age levels, from kindergarten to primary school levels. Data from the reviewed studies will contribute to identifying major risk factors and domains of difficulty, connected to later academic success. Given the increasing number of late preterm children in our society and the limited number of resources available to systematically implement follow-up protocols in this population, the review can contribute to highlighting potential screening strategies to facilitate early risk detection and to develop guidelines for intervention to minimize the effects of this morbidity in childhood. More generally, our specific contribution with the present review is to offer an updated and more nuanced perspective on the still existent controversial assimilation of healthy late preterm and term-born populations regarding their risk for neurocognitive difficulties and low academic achievement.

## 2. Materials and Methods 

### 2.1. Data Source and Search Strategy

The current systematic review followed the Preferred Reporting Items for Systematic Review and Meta-Analysis (PRISMA) statement [34]. Studies were first identified by independent searches performed by each author early in year 2020 using several computerized databases, including pubMed, Scielo and PsycINFO. The search was restricted to academic publications in English from January 2010 to May 2020. The following search terms were used in combination to refine the initial search based on two broad descriptors (premature birth AND late preterm): AND “outcomes” AND “school/education/academic performance/achievement” OR “cognitive function/outcomes” OR “general cognitive ability” OR “developmental outcomes” OR “intelligence” OR “learning” OR “attention” OR “attention-deficit/hyperactivity disorder” OR “working memory” OR “child development” OR “follow-up” OR “assessment” AND “school age”. Then the authors analyzed titles, abstracts, and full texts to find eligible studies and also checked the papers from the list of references in the selected articles.

### 2.2. Selection of Eligible Studies: Inclusion and Exclusion Criteria

The inclusion criteria applied to studies for this review were: (1) a comparison between healthy late preterm children and a term-born control group on assessments performed at 3 years of age or beyond (longitudinal studies with assessments beginning at earlier ages were included if they also reported results at 3 years of age or beyond); (2) representative samples of participants, excluding studies on cohorts born in the 80s or early 90s that were already described in previous reviews; (3) studies reporting measurements on learning outcomes, cognitive functioning and/or language communication performance at any point in time from 3 years of age (during pre-school, kindergarten and/or school years), involving either a single age level or a longitudinal approach; (4) studies reporting data from a broader preterm group (moderate-to-late preterm) were only included if they separately reported results from the late preterm group or if mean gestational age fell into the late preterm range and the sample was representative; (5) published in an English language, peer-reviewed journal. Papers that did not meet these inclusion criteria were excluded. Furthermore, reviews, systematic reviews, and meta-analysis were also excluded.

**Figure 1:**
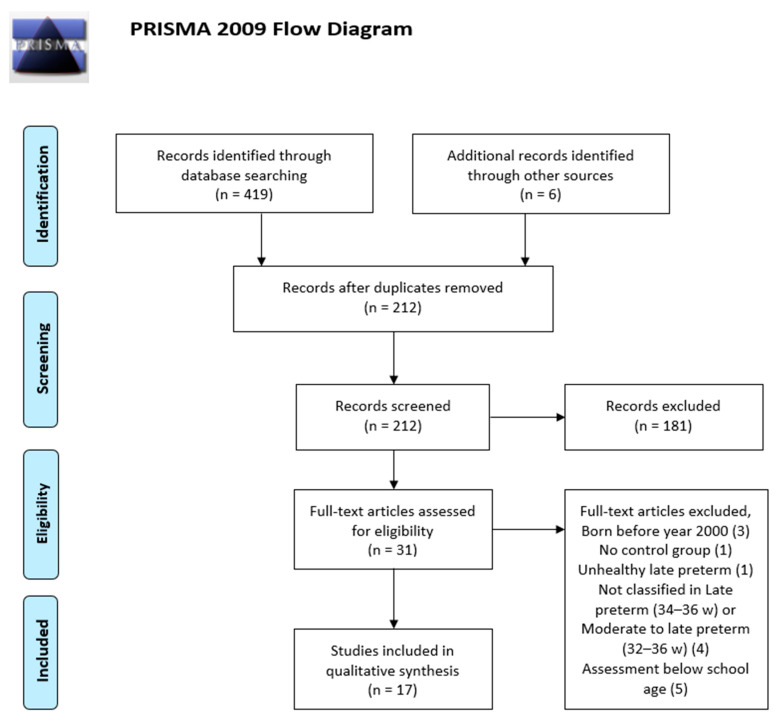
Flowchart showing the study selection process.

Based on screening of titles and abstracts, from the initial search results 212 articles remained and were further assessed for eligibility. A total of 182 records had to be excluded based on the following criteria: not meeting any of our inclusion criteria relative to age range, degree of prematurity, behavioral assessment only, review and systematic review papers (28); studies reporting different gestational problems (preeclampsia and placental alterations, intrauterine growth restriction, in vitro fertilization effect, predictors of preterm delivery, maternal drugs, maternal metabolic profile, a range of maternal diseases -heart, dental and oncologic problems, malaria, thalassemia major- and maternal surgery) (39); delivery conditions (timing of umbilical cord clamping, cardiac assessment during the extra uterine transition) (7); postnatal morbidity (perinatal infection, neonatal morbidity with or without malformations, postnatal corticoid treatment and long-term respiratory morbidity) (45); studies focusing on neonatal needs (feeding/nutrition, probiotics, family-centered developmental care in the NICU) (37); studies on a number of different topics not within the focus of our review (epidemiology of prematurity, socio economic factors related to outcomes, early intervention in the child and/or mother, rotavirus vaccine, age of schooling onset and performance, brain MRI in late preterm and very preterm) (26). A total of 16 studies were found suitable as they met our inclusion criteria, reporting data on the neurodevelopmental follow-up of late preterm or moderate-to-late preterm children at age 3 years or beyond, so they were selected and included in the analysis. The flow diagram of study selection is presented in Figure 1.

### 2.3. Data Extraction

With the selected articles we first grouped them into two broad categories relative to a) studies whose main focus was measuring cognitive functioning, and b) those reporting objective measures of academic performance. For each of these two categories data relative to the following aspects were extracted: authors, year of publication and location; design of the study; ages at which assessment took place; characterization and size of the late preterm sample; control group sample; main aim of the study; modality of assessment undertaken (tools used, observational and/or experimental approaches) and adjustment for possible confounders; main results. These data constitute the core of the present review and were carefully compiled for analysis and interpretation.

### 2.4. Quality Assessment

One of the authors (SM-N) assessed the methodological quality of the selected articles using the Critical Appraisal Skills Programme (CASP, Oxford Centre for Triple Value Healthcare Ltd., Oxford, UK) checklist for cohort studies [35]. The checklist includes a first section with six global aspects (eight questions overall) to specifically assess the validity of the results of the studies. The checklist has two initial questions that need to obtain a positive answer in order to continue the analysis, namely clear focus of the study and recruitment procedures. The remaining questions address the following aspects: measurements and participant classification biases; outcome measurements and bias control; identification and control over confounding factors; and follow-up characteristics related to its completion, significant loss of participants possibly affecting the outcomes and length of the follow up (e.g., time distance between assessments sufficient to reveal stability or change in the outcomes). Due to the rather limited age range of the participants in the selected studies, we considered as positive follow-up assessments those conducted at two different time points during the pre-school and school years (longitudinal data), but also those conducted only at one time point beyond three years of age. From the checklist a maximum score of 8 could be obtained (score 1 for each positive answer and 0 for a negative or unclear answer).

## 3. Results

In this section we describe the main findings after extracting the information from the selected papers for review that met our inclusion criteria. The selected studies, most of them reporting neuropsychological assessments including cognition and language measurements, and just a few of them centered on academic performance, have been grouped into a single table ordered according to assessment age (see Table 1). The studies are described taking into account the nature of the samples, their methodological characteristics and the main findings related to the late preterm children vs. term-born comparison, even though some of the studies involved additional comparisons with other preterm groups of a lower gestational age. The type of assessment tools used (e.g., developmental scales, standardized tests, experimental tasks, parental questionnaires, etc...) is also reported so as to gain a general perspective on the ones most frequently used and to discuss their specific contribution.

### 3.1. Included Studies

From the initial database searches and after excluding duplicates as well as the non-eligible studies following the criteria reported in Section 2.2 (see also Figure 1), sixteen studies met inclusion criteria, thirteen of them evaluated neuropsychological outcomes taking into account cognitive and language functions and only three had the main focus on academic performance. The main factor determining exclusion of studies was the absence of a well-established late preterm (LPT) group, with data separated from data corresponding to other preterm groups with lower gestational ages. However, some studies offering data from a broader preterm group (moderate and late preterm, MLPT) were included if they separately reported results from the LPT group, or if the mean gestational age fell within the late preterm range and the sample was representative. As for the age range, studies were included if they reported measurements obtained at age 3 years or beyond, whether longitudinal or not. This criterion has been followed strictly so studies reporting data from a sample of three-year-olds, as well as longitudinal follow-up studies whose second assessment took place at three years of age, have been included.

### 3.2. Description of Included Studies

As just mentioned, selected studies have been grouped into a single table ordered by assessment age, from younger to older participants’ data (see Table 1 for a summary of the main features and findings).

We will begin by describing the subset of studies addressing neuropsychological outcomes, in which findings related to cognitive functioning and language outcomes are included. Then we will describe the small subset of studies specifically reporting data on school performance from standardized measurements of academic achievement. The focus of the assessment (neurocognitive skills vs. academic achievement) and the nature of the assessment tools clearly differs between these two types of studies and justifies a separate description, however these two domains are undoubtedly interconnected, with cognitive and language skills affecting academic performance. This issue will be further considered in the discussion.

#### 3.2.1. Neuropsychological Outcomes: Cognitive Functioning and Language

Thirteen studies are grouped in this category and they have been developed in the US (7), Canada (2), Norway (1), The Netherlands (2) and Denmark (1). All studies involved healthy LPT, that is, late preterm children without neonatal compromise. Studies tackle different aspects of cognitive functioning, namely, executive functions, EFs (4); verbal inhibitory control and short-term verbal memory (1); visuomotor integration and visuospatial construction (1); processing speed and attention (1) or memory (1). Other studies adopt a more general measure of neurodevelopment (3). These different areas are also being explored by means of different measuring tools. Typical experimental laboratory tasks are mainly used for measurements of EFs, adapted to the age of the participants. Neurodevelopmental levels are assessed by means of standardized scales or tests, such as Wechsler Preschool and Primary Scale of Intelligence (WPPSI) or Peabody Picture Vocabulary Test (PPVT) or, alternatively, by parental questionnaires such as the Ages and Stages Questionnaire (ASQ). A more detailed description of the tools for assessment and results obtained is offered below, grouping the information according to the age of the participants in the studies. 

The assessment tools for 3-year-olds were: different tasks and tests of EFs, as the Preschool Continuous Performance test (P-CPT); Boy-Girl Stroop; Go/No-Go and Jack’s Boxes to assess working memory and response inhibition [36]. The Ages and Stages Questionnaire was used to assess five domains of development (communication, gross motor, fine motor, problem solving and personal social skills) [37]. The Motor and Social Developmental Scale (MSD) was included to evaluate whether the child performed in age-appropriate behaviour [38]. In these evaluations late preterm children showed worse results than term-born children despite adjusting for different confounders. Baron et al [36] found that late preterm children performed worse on complex working memory tasks, but not on response inhibition measures.

From 4 to 5 years of age, the assessment included a battery of tests, inventories and/or EF tasks: the Peabody Picture Vocabulary Test (PPVT) that measures receptive vocabulary and offers an estimate of verbal ability or scholastic aptitude [38,39,40]; the Behaviour Rating Inventory of Executive Function in the preschool version (BRIEF-P) which assesses eight domains and offers the global executive function composite [39,41]; the Preschool Comprehensive Test of Phonological and Print processing (Pre-CTOPP) useful to assess three areas of phonological processing, i.e. phonological sensitivity, memory, and access [40]; Hot and Cool EF tasks that assess, respectively, self-management skills when emotions run high (social cognition, emotion regulation and decision making) and when emotions are not involved (e.g., planning, cognitive flexibility, working memory, initiation, suppression and concept formation) [42].The WPPSI offering IQ scores at preschool age [41,42]. The Ages and Stages Questionnaire (parental questionnaire) was also used at this age range [43] and the Test of Everyday Attention for Children (TEACh) was used to evaluate attention capacities [41].

Results obtained at this age level show verbal alterations, in verbal inhibitory control and in short-term verbal memory [39]. Late preterm also differ from full term children in pre-reading skills at 4–5 years, showing a less optimal outcome that continues to be found by age 6 years [40]. Hornman et al. [43] evaluating the stability of developmental problems by means of the Ages and Stages Questionnaire found an abnormal total score in 7.9% of MLPT children at 4 years, a significantly higher percentage than in full-term children (4.1%). However, one year after school entry, the overall Ages and Stages Questionnaire score was comparable between both groups (MLPT and full-term children). Brown et al. [38] found a receptive vocabulary delay at 4 to 5 years higher in LPT than in full-term children, although when controlling for confounder factors these results were no longer significant. Sejer et al. [41] did not find an association between lower intelligence and poorer EFs in a late preterm vs. full-term comparison. With respect to Hot EF tasks at preschool age, MLPT children were less likely to choose the larger, delayed rewards across all levels of reward magnitude on a delay discounting task, in comparison to full-term children; but no between-group differences were found on measures of Cool EFs [42]. 

At 6 years of age the tests performed to assess EFs (the modified Baron-Hopkins Board test) explored five indicators of EFs (noun fluency, action-verb fluency, similarities reasoning, matrices reasoning and working memory) [45]. The Differential Ability Scales (DAS) was used to assess general cognitive abilities yielding verbal, nonverbal reasoning, spatial cluster scores and a general conceptual ability (GCA) score [45,46]. Studies also include tests to evaluate visuoconstruction perception (TVSC) and the integration of visuoperceptual ability and fine motor coordination [46]. Baron-Hopkins Board test (B-HB) evaluates the recall of monochromic pictures [46], and Purdue Pegboard Test of Manual Dexterity assesses motor dexterity and coordination [46]. To evaluate global cognitive function the WPPSI and Child Behaviour Checklist (CBCL) were used [47].

As for the results, Baron et al. [45] and Rider et al. [46] found better results in EF tasks at 6 years compared to results at 3 years. By age 6 only trivial or subtle differences were found, but Bogicevic et al. [47] examining cognitive function found poorer processing speed and more attention problems in MLPT children compared to the control group. It was also found that poorer alerting and orienting of attention skills, previously reported at 18 months of age, were identified as precursors for later lower performance IQ and attention problems, respectively.

For older children beyond 7 years of age studies used the Grooved Pegboard Test (GPT) assessing eye-hand coordination and motor speed, thus requiring sensory motor integration and high level of motor processing [51]; Physical and Neurological Examination for Soft Signs (PANESS) was used to assess laterality and timed and untimed motor movements [51]; Monkey Tree Task (MTT), an adapted version of the Tower of Hanoi test, was used to assess executive attention [50]. Cognitive function and general intelligence quotient were obtained using the Wechsler Intelligence Scale for Children (WISC) [51]. Only 2 studies involving 7-year-old or older participants met inclusion criteria and could be included in the review. Brumbaugh et al. [51] assessed children at 6 and 13 years of age and found slower processing speed, poorer visual-spatial perception and memory in late preterm children compared to full-term participants. Although parents referred more behavioural difficulties, there were no group differences in cognitive ability or academic achievement between LPT and FT groups. However, neuroimaging studies revealed between-group differences, with late preterm showing less total brain tissue, more cerebrospinal fluid and smaller thalami compared to term-born children. Sheehan et al. [50] on the other hand found that early differences between LPT and FT in planning skills detected at age 3 years were no longer present at 6 and 9 years.

Finally, it is worth pointing out the limited number of studies centered on communication and language, which is surprising because these are areas clearly connected with learning and academic achievement. Stene-Larsen et al. [37] found that late preterm had an increased risk for communication impairments at age 3 years. For assessments al later ages, around 4 to 6 years, two studies report results more connected to the academic performance in preschoolers: preschool reading and preschool mathematics, as well as kindergarten reading were assessed in Shah et al. [40], while Brumbaugh et al. [51] evaluated academic achievement in older children using the Wide Range Achievement Test (WRAT) that measured reading, spelling, comprehension and math skills. The former found significant differences between LPT and FT at preschool age, while this less optimal performance was just limited to reading once in kindergarten (5–6 years). On the other hand, Brumbaugh et al. [51] did not find significant differences in cognitive ability or academic achievement between late preterm and full-term children. 

An overview of the abovementioned results reveals a complex and rather heterogeneous output. While in some of the areas late preterm perform below the level attained by the full-term counterparts, especially at earlier ages, in the second assessment and in some tasks and measurements late preterm seem to catch up. This pattern was observed in the visuomotor domain and in some EF tasks. A similar pattern was also obtained when assessing general cognitive function, either in studies using parental questionnaires or in the ones using tests and scales administered by a specialist. Among those areas that tend to remain less optimal, processing speed and attention remain affected by age 6, levels in language and pre-literacy skills are also lagging behind the levels obtained by term-born children, hot EF measures (but not cool EF measures) are also suboptimal by age 5, and visuospatial perception and memory are still poor when assessed by 13 years of age. 

Non-convergent results could be a consequence of the different methods and tools used in each study. Sometimes the differences are subtle, not reaching significance, but nevertheless the authors express their concern and suggest that the population should be considered at risk. Interestingly enough, the influence of social factors, such as maternal education and socio economic status, on the results by late preterm participants is highlighted, suggesting that rather than the gestational age factor per se, it is a combination of factors, some of them in the social domain, which can negatively affect the catch up process in the late preterm population. 

#### 3.2.2. Academic Outcomes

Studies specifically addressing measurements of academic performance and school outcomes in late preterm participants were scarce in our selection. Out of three studies, two were developed in the UK [44,49] and one in the US [48]. These three studies deal with different age levels (5, 4–6 and 7 years, respectively) and academic performance was evaluated according to the specific country or local region standards. In the UK, we found two options for evaluation: a) the Foundation Stage Profile (FSP) that assesses personal, social and emotional development, communication, language and literacy goals that is applied in government-maintained (state) schools and independent (fee-paying) schools who receive government funding [44]; and b) Key Stages which begins in school year 1 (age 5–6 years) and is completed by the end of year 11. Key Stage 1 (KS1) is applied to evaluate 5–7 year-olds’ achievement in five key domains: reading, writing, speaking and listening, mathematics and science in all state-funded and some private schools [49]. In the US at kindergarten year, Total School Readiness Score (TSRS) is a cognitive battery that includes reading, math, and expressive language testing [48].Academic performance outcomes in late preterm, but also in moderate preterm children at 5 years of age tested with Foundation Stage Profile (FSP) at the end of the child’s first school year, revealed their higher risk for suboptimal academic achievement, as they did not reach the expected level of performance [44]. In the US study developmental outcomes from infancy to kindergarten were compared [48]. Children were evaluated with Total School Readiness Score (TSRS) and LPT showed worse results than full-term. Chan et al. [49] referred poor performance at 7 years using the KS1; late preterm and also moderate preterm birth should, thus, be considered factors that increase the risk for poor school performance, especially when added to other risk factors such as gender, parental education or school attendance. 

In sum, all three studies converge in reporting higher risk for poorer academic performance in late preterm compared to full-term children. In these studies several confounding factors were taken into account and results were adjusted for them, so the main picture obtained from the still limited studies in this domain supports the notion that late preterm cannot be easily assimilated to early term or full term groups.

### 3.3. Quality of Papers

The Critical Appraisal Skills Program (CASP) checklist was applied to assess the methodological quality of the selected papers in this review. The overall quality was found to be satisfactory (see Table 2). All papers obtained a positive answer to the first two screening questions, so it was worth proceeding with the remaining questions. The total of positive answers ranged from five to eight, out of the eight aspects being considered. In the majority of studies the number of children assessed was sufficient, except in those studies addressing measurements of executive function by means of experimental laboratory tasks that were based in small samples. Confounding factors were not identified and taken into account in the design and/or analysis in several studies, mainly those assessing executive function. As for the follow up, it was considered complete enough only in nine papers. But, on the other hand, it was reasonably long in fourteen of the studies, involving measurements at two different time points corresponding to non-consecutive years, thus offering some perspective on the stability or change of the differences between late preterm and full term children in specific areas of their cognitive and language development.

## 4. Discussion

In this paper we have offered a systematic review of recent data, based on selected publications in the last decade, assessing late preterm children outcomes mainly in the cognitive functioning domain, the language domain and in academic achievement. A final set of 16 articles reporting data from healthy populations of children born late preterm, assessed at some point during preschool and primary school levels, at or beyond the age of 3 years. We decided to limit the review of studies addressing LPT cognitive and learning outcomes to those covering these early school years, that is, excluding papers centered exclusively on younger participants up to 24 months of age. It has been reported in the literature that some of the early developmental differences between LPT and FT control groups identified in the first year of life are no longer present by age 2 years, suggesting that late preterm infants gradually catch-up. It becomes then relevant to review data from age 3 and beyond in order to better capture the nature of these early catch-up processes, their stability and the changes that can occur when the cognitive demands increase as children face more complex learning situations in pre-school and school contexts. As suggested in previous reviews, late preterm birth should be viewed as a risk factor for poorer academic performance. A closer look at late preterm-full term comparisons to identify less optimal performance in specific areas of the cognitive and language domains along the early school years can reveal a more nuanced picture of the effects of late preterm birth on long-term academic outcomes. More studies were found addressing cognitive functioning compared to studies assessing language or academic performance outcomes. The results are complex and heterogeneous, but they continue to reveal a trend towards the existence of mild difficulties in this population, significantly differing from full-term children in several of the measurements, especially those relative to executive function, attention, processing speech, working memory, and literacy and mathematics in the academic domain. Differences are often subtle and some of them no longer present once at school age, while others evolve but persist in time, often compromising successful academic achievement.

Certainly, more research is needed in this domain, but the debate on the assimilation of healthy late preterm and full-term children regarding their similar risk for neurocognitive difficulties and low academic performance unfortunately remains unsettled. So far the results suggest the advisability of including this low risk population in regular screening and follow-up programs, so as to have an early identification of those with higher risk for learning difficulties and implement intervention strategies to promote a better academic achievement. 

Data on this population remains limited, even though late preterm constitute the larger subset of all preterm infants. Moreover, in order to gain a better understanding of their cognitive risk, studies addressing late preterm and moderate-to-late preterm populations explored beyond early childhood should be planned, but the number is still limited. Previous systematic reviews and meta-analyses can be found (e.g., McGowan et al. [31], Tripathi and Dusing [32], Chan et al. [33]), involving cohorts of children born in the 1990s or even earlier and differing in the scope of the age range considered. In spite of the differences, those reviews converge in their conclusions about late preterm children being at increased risk of adverse developmental outcomes and academic difficulties in comparison to term-born children. LPT-FT differences seem to be especially consistent in the cognitive domain of neurodevelopment, although they are also present in the language domain, readily connected to academic performance. Differences, although small and even subtle, continue to be identified even after controlling for social factors known to have adverse effects on school performance. Previous reviews also emphasize the need for more focused follow up research to further analyze the adverse effects of late preterm birth on childhood development. It is interesting to note that a couple of studies in Chan et al. review [33] report data on cohorts of participants that were followed up and results at later ages are presented in papers included in our review [44,51], thus offering the longitudinal perspective that reveals maintenance of some cognitive and academic performance differences between LPT and FT samples.

The present systematic review is, to the best of our knowledge, the first to analyze cognitive and learning outcomes in cohorts of recently born children, most of them born after the year 2000. It is informative about possible differences from earlier studies with older cohorts, when prenatal care of women was less optimal and family-centered developmental care had not yet been established in the Neonatal Intensive Care Unit (NICU). It also shows the range of tools that can be used to assess this population beyond gathering global measures on their cognitive, language and motor functioning form standardized developmental scales or tests yielding an intelligence quotient measure. The conceptualization of attention and executive functions has enabled the development of a number of laboratory tasks and techniques that can shed light on more subtle mechanisms underlying the overt behaviour of participants in these studies. What to evaluate and how to evaluate are central issues to the still open question about the “negative” impact of late preterm birth on neurocognitive development. A closer look at the assessment tools used in the studies that have been reviewed reveals a broad range of tests and tasks that, as expected, differ between countries and vary according to the age of the participants. However, three broad categories of tools can be identified, fulfilling somewhat different assessment functions. First, standardized scales or tests that enable to identify the level of attainment compared to normative data, but often lack the capacity to obtain a more narrow perspective on the underlying mechanisms supporting the specific performance of participants in different subtests related to different cognitive domains. These are adequate tools for general between-group comparisons and screening purposes. Parental questionnaires, even if extensive ones such as the ASQ, very often used in the reviewed studies, are also adequate for screening purposes, so as to gather neurodevelopmental data on different domains, but they cannot offer this more nuanced perspective on the cognitive functioning of the children when facing specific tasks, problems or novel situations. This is why laboratory tasks or "problems", such as the ones used to assess executive attention, working memory and control mechanisms, seem suitable to complement the range of assessment tools available. Measurements of online processing of the information, implemented in a clinical setting, might contribute to reach a better understanding of the connections between cognitive functioning and academic performance in the LPT population.

A clear explanation for the complex and sometimes fluctuating pattern of results described in this paper has not yet been provided. The catch up processes described in some skills or tasks seem to be transient and disappear with new assessments at later ages. Task demands must then be taken into account in order to explain some of the gains and delays in performance. One factor that deserves further attention is the role of the environment on learning outcomes. Social factors that are considered relevant and fundamental to successful language learning should also be incorporated in future research and be better explored regarding the learning processes and outcomes of late preterm children. Beyond socioeconomical status (SES) and parental education factors, we want to emphasize here the role of social interactive factors, those that affect learning by favouring, enhancing it through adequate interaction patterns between the young learner and the adult/s in her environment. Early triadic interaction contexts in which joint attention processes are taking place and timely adult responsiveness is present facilitate learning, especially in the language acquisition domain. The social interactive processes continue to favor learning, not just restricted to language, at later developmental ages and this is an issue that is awaiting more systematic research in the context of at risk populations such as LPT children. 

There is also some evidence that preterm infants with neonatal morbidity have worse outcomes than those term-born and those healthy preterm of similar gestational age [11,12,52,53,54]. This can be a confounding factor when exploring healthy late preterm outcomes. For this reason, we excluded studies with heterogeneous samples and only included studies involving healthy late preterm or moderate-to-late preterm children. As a consequence, results are more limited, restricted to a small number of studies. Only nine of the selected studies took into account and adjusted for a number of confounding factors, predominantly those related to the family environment and specifically related to maternal education, age, SES, marital status, smoking, alcohol consumption, prenatal care and delivery type. The fact that confounder factors are not always reported represents another limitation in the interpretation of the data.

Having narrowed the scope of our systematic review to publications in the last decade, many of them assessing more recent LPT cohorts than those included in previous reviews, involves an implicit limitation as it constrains the possibility of having data from older children, beyond 8–9 years of age. Results from studies including older LPT children would help confirm or disconfirm the stability of between-group LPT-FT differences already described in younger participants. Therefore, the developmental risk of late preterm or moderate-to-late preterm children without neonatal morbidity needs to be further assessed, with studies including a better control of confounding factors and longer follow up measures in order to reach more robust conclusions about the different factors affecting learning and academic outcomes, and their relative weight. The design and implementation of early detection protocols based on this knowledge would improve the identification of children with higher risk for less optimal learning outcomes and school performance.

It is important to keep in mind the connection between sometimes subtle cognitive functioning differences, evidenced in late preterm vs. full-term comparisons, and their possible impact on later academic performance. Attention and executive function are areas in which mild differences or difficulties have recurrently been described in late preterm children who had otherwise obtained an acceptable result in more general measurements of cognitive development. The differences detected in complex executive function tasks could be related to poorer academic performance. The sensitivity of the tasks used to assess executive functions at specific age levels is important, as we have seen difficulties appearing at preschool age, but disappearing in kindergarten and beyond. More research is needed in this important research domain to better delimit the scope and nature of the academic difficulties and their connection with cognitive processes and mechanisms that might be affecting learning in a subtle but persistent way.

## 5. Conclusions

Late preterm or moderate-to-late preterm children can be considered as an at risk population for neurocognitive difficulties that might negatively affect learning and academic performance in the long term. Reviewed data from recent studies involving measurements of cognitive functioning, language and academic performance reveal a complex and changing pattern of difficulties, especially evident in the executive function domain, attention, processing speed, visuospatial perception, working memory and language skills that might underpin reading and maths learning difficulties once at school. Moreover, the reported differences between late preterm and full-term control groups have turned out to be subtle, some of them transient, but nevertheless detectable by means of specific experimental tasks and likely to be connected to late preterms’ poorer academic results. Future work needs to refine the assessment procedures, apply them to larger samples and extend follow up studies so as to eventually develop effective screening strategies that should facilitate early risk detection and minimize the negative effects of this morbidity in childhood.

## Figures and Tables

**Table 1:** Neuropsychological and academic performance outcomes: Characteristics of the selected studies in these domains, where cognitive and language functions are included. Studies have been ordered by main age of the assessment, starting from age 3 years. The results column includes a short synopsis specifically highlighting the main findings related to late preterm groups’ performance.

Authors, Year and Location	Study Design/ Year of Birth	Study Population	Primary Objective	Assessment Ages/Tests or Tasks/ Confounders Adjusted for	Results/ Short Synopsis
Baron et al., 2012.US [36]	Longitudinal cohort study from PETIT (Prematurity’s Effects on Toddlers, Infants and Teens) Born 2004–2006	ELBW n = 52 LPT n = 196 Control group FT n = 121	To compare ELBW, LPT and FT children with a FT control group on a novel battery of experimental computerized EF tasks.	3 years Selected EF tasks: -P-CPT -Boy-Girl Stroop -Go/No-Go -Jack’s Boxes -DAS Confounders: GA. Maternal educational level	LPT < FT on complex working memory. LPT = FT on response inhibition measures. LPT < FT on General conceptual ability (GCA) LPT > FT on omission errors in the P-CPT task **Selective EF tasks can distinguish between preterm groups of different GA and FT children in preschool years. LPT performed worse than FT on complex working memory.**
Stene-Larsen et al., 2014. Norway [37]	Cohort study from Norwegian Mother and Child Cohort Study Born 1999–2008	LPT n = 1673 ET n = 7109 Control group FTn = 30,641	To investigate the risk of communication impairments at age 18 and 36 months in children born ET and LPT.	18 months and 3 years -ASQ (Questionnaire) Confounders: Child gender, maternal age, maternal level of education, maternal gestational diabetes, preeclampsia/HELLP syndrome, multiple gestation, SGA.	LPT (and ET): increased risk of communication impairments at both ages. -Communication impairment (18 months): ET aRR 1.27 (95%CI 1.12–1.44), LPT aRR 1.74 (95%CI 1.41–2.14) -Expressive language impairment (36 months): ET aRR 1.22 (95%CI 1.07–1.39), LPT aRR 1.37 (95%CI 1.09–1.73) **LPT are at increased risk for communication impairments. Given the large number of children potentially affected, this may result in significant health care costs.**
Brown et al., 2014. Canada [38]	Cohorts study Secondary analysis of National Longitudinal Survey of Children and Youth (NLSCY) Born 1994–2009	2 to 3 years LPT n = 1102 ET n = 4333 4 to 5 years LPT n = 866 ET n = 3478 Control group FT 2 to 3 years N = 9664 4 to 5 years N = 7859	To elucidate the role that GA plays in determining risks for poor developmental outcomes in LPT and ET in the context of proximal social processes	2–3 years and 4–5 years - 2–3 years: MSD - 4–5 years: PPVT-R - Parenting Scale Confounders: Smoking, alcohol use during pregnancy, placental ischemia and other hypoxia, maternal diabetes or other medical condition during pregnancy. Social context as described in terms of family structure. Family resources and family functioning. Child gender.	2–3 years: LPT > FT rate of developmental delay (16.7% LPT, 13.9% FT). LPT: aRR 1.13 (95%CI 0.90-1.42) 4–5 years: LPT > FT rate of receptive vocabulary delay (13.1% LPT, 12.7% FT) LPT: aRR 1.06 (95%CI 0.70–1.43). **LPT closer to FT. Social factors (not GA) maybe the most important factor influencing developmental outcomes**
Brumbaugh, et al., 2014.US [39]	Prospective cohort study Born 2005–2006	LPT n = 39 Control group FT n = 44	To assess whether LPT children demonstrate impaired EF compared with full term children	4 years -Battery of EF tasks -PPVT -BRIEF-P Confounders: Verbal IQ, GA	LPT < FT on verbal inhibitory control and short term verbal memory tasks. LPT = FT on nonverbal inhibitory control or spatial memory. Parents of LPT and FT rated children’s behaviour similarly. GA as a predictor of the task performance. **LPT demonstrated compromised verbal inhibitory control and short-term verbal memory compared with full-term peers. LPT may not be spared from altered brain development.**
Shah et al., 2016. US [40]	Cohort study Early Childhood Longitudinal Study, Birth Cohort (ECLS-B) Born 2001	LPT n = 1000 Control group ET n = 1800 FTn = 3200	To compare developmental outcomes of LPT with ET and FT from infancy to kindergarten	9 and 24 months, 3 years, 4–6 years -9 and 24 months: BSF-R -Preschool-Kindergarten: -PPVT -Pre-CTOPP -Preschool pre-reading assessment and mathematics Confounders: -Maternal age, maternal race or ethnicity, socioeconomic status at 9 months, parenting, infant gender, birth weight, early intervention services -At preschool and Kindergarten: age at assessment, month of school	-9 months: LPT < FT (and ET) in developmental outcomes (T = 47.31) vs. ET (T = 49.12) and FT (T = 50.09). -24 months: LPT = FT in developmental outcomes -Preschool age (3 years): LPT < FT in pre-reading skills and mathematics. -Kindergarten (4–6 years): LPT < FT in reading. **Although LPT seem to catch up and demonstrate comparable developmental outcomes to FT at 24 months, later on they demonstrate less optimal pre-reading and reading skills and maths at preschool and kindergarten time points.**
Sejer et al., 2019. Denmark [41]	Cohort study Lifestyle During Pregnancy Study (LDPS) Born 2003–2008	VPT to MPT n = 8 LPT n = 40 Control group FT n = 1728	To assess the impact of GA on intelligence, attention and executive function at 5 years	5 years -WPPSI-R -TEACh -BRIEF Confounders: Maternal age at birth, maternal IQ, average alcohol consumption in pregnancy, smoking in pregnancy, parity, maternal marital status, parental educational level, child gender.	Very to moderate preterm obtain -10.6 IQ vs. full term and -5.3 in teacher-assessed Global Executive Composite, adjusted results. No association with poor cognition were shown in LPT. **No associations between LPT and poor cognitive outcomes were shown at age 5. GA may play an important role in determining cognitive abilities independent of maternal intelligence and parental education.**
Hodel et al., 2016. US [42]	Observational cohort study Born: NA	MLPT n = 45 Control group FT n = 46	To determine whether low-risk, healthy children born MLPT also exhibit impairments in the development of prefrontal-dependent hot EF skills in comparison to term children at preschool age.	4.5–5 years -Hot EF tasks -Cool EF tasks -WPPSI-III -BRIEF-P Confounders: Intelligence, processing speed	M-LPT at age 4.5 years< FT less likely to choose larger, delayed rewards across all levels of reward magnitude on a delay discontinuing task using tangible rewards. MLPT = FT on a delay aversion task involving abstract rewards and on measures of cool EFs. **Evidence of disrupted hot EFs in children born MLPT at preschool age as measured on a delay discontinuing tasks.**
Hornman et al., 2017. Netherlands [43]	Cohort study Longitudinal Preterm Outcome Project (LOLLIPOP) Born 2002–2003	EPT n = 376 MLPT n = 688 Control group FT n = 403	To assess the stability of developmental problems before school entry at 4 years and one year after school entry at 5 years	4 and 5 years -ASQ Confounders: Sex, SGA, multiple birth, low education level of the parents, non-Dutch birth country of parent or children, single parent family	4 years: MLPT < FT 7.9% (*p* = 0.016); EPT 13% (*p* < 0.001), FT 4.1% 5 years: MLPT = FT On underlying domains, MLPT and EPT had mainly emerging motor problems and resolving communication problems, but the changing rates of MLPT were lower. **After school entry, the overall development of MLPT shows stability patterns comparable with FT. On the underlying domains, MLPT had patterns comparable with EPT but lower rates.**
Quigley et al., 2012. UK [44]	Cohort study Millennium Cohort Study (MCS) Born 2000–2002	VPT n = 84 MPT n = 92 LPT n = 471 ET n = 1596 Control group FT n = 5407	To compare school performance at age 5 years in four groups of preterm children differing in GA (ET, LPT, MPT and VPT) and a FT control group.	5 years -FSP Confounders: Child gender, ethnicity, whether firstborn, breastfeeding duration, month of birth, mother’s age at, delivery, marital status, education, social class, languages spoken in the child’s home	% not reaching a good level of overall achievement: -FT: 51% -ET: 55% aRR 1.05 (95%CI 1–1.11) -LPT: 59% aRR 1.12 (95%CI 1.04–1.22) -MPT: 63% aRR 1.19 (95%CI 0.98–1.45) -VPT: 66% aRR 1,19 (95%CI 1–1.42) **LPT birth is associated with an increased risk of poorer educational achievement at age 5 years.**
Baron et al., 2014. US [45]	Retrospective observational cross-sectional cohort study from PETIT (Prematurity’s Effects on Toddlers, Infants and Teens) Born 2000–2009 3 years assessment: Born 1998–2006 6 years assessment:	3 years -ELBW n = 93 -LPT n = 398 Control group FT n = 177 6 years -ELBW n = 126 -LPT n = 102 Control group FT n = 183	To use latent means analysis in structural equation modeling (SEM) to make between-group comparisons (ELBW, LPT and FT) in EF at two time points: preschool (3 years) and early school age (6–7 years)	3 and 6 years - Baron-Hopkins Board Test -DAS EF indicators: 1.Noun fluency 2.Action-verb fluency 3.Similarities reasoning 4.Matrices reasoning 5.Working memory Confounders: No	3 years: LPT < FT (0.61 SD). 6 years: LPT = FT (0.10 SD) Statistically significant between-group differences at age 3, but no longer present at age 6. **LPT showed higher risk for EFsdeficits than FT at an early age. Deficitscould represent a transient developmental delay likely to resolve at an older age, or a more subtle adverse effect likely to persist over the life span.**
Rider et al., 2016. US [46]	Retrospective cohort study from PETIT (Prematurity’s Effects on Toddlers, Infants and Teens) Born 3 years assessment: 2006–2010 6 years assessment: 2004–2007	3 years -ELBW n = 53 -LPT n = 228 Control group FT n = 74 6 years -ELBW n = 42 -LPT n = 141 Control group FT n=82	To provide convergent validity evidence for TVSC To examine performance differences between participants born ELBW, LPT or at FT at preschool (3 years) and early school age (6 years)	3 and 6 years -TVSC -Developmental Test of Visual-Motor Integration (VMI, 5th ed.) -DAS-II -Baron-Hopkins Board Test -Purdue Pegboard Test of Manual Dexterity Confounders: No	3 years: LPT < FT 6 years: LPT = FT **TVSC practical differences between LPT and FT were small at age 3 and trivial at age 6 years. Although LPT at 6 years performed comparably to FT on the TVSC, LPT should not be considered absent of risk.**
Bogicevic et al., 2019. Netherlands [47]	Prospective cohort study, Study in Attention of Preterm children (STAP Project) Born 2010–2011	MLPT n = 88 Control group FT n = 83	To compare cognitive and behavioural functioning at 6 years. To assess which toddler skills predict later cognitive and behavioural functioning	18–24 months and 6 years 18 months: UTATE. 24 months: Bayley-III-NL 6 years:WPPSI-III-NL; CBCL/6-18 Confounders: Maternal education	- MLPT < FT on processing speed - MLPT > FT on behavioural problems. -At 6 years: Attention problems were predicted by poorer orienting of attention skills at 18 months and lower performance IQ was predicted by lower alerting of attention at 18 months. Full scale and verbal IQ were predicted by language skills at 24 months. **Poorer functioning in MLPT at primary school-age reveals vulnerabilities specifically in processing speed and attention problems, which suggests the need for specific assessment of these skills. Poorer orienting of attention skills at toddler age as early predictors of later attention problems.**
Woythaler et al., 2015. US [48]	Cohort study Early Childhood Longitudinal Study, Birth Cohort (ECLS-B) Born 2001	LPT n = 950 Control group Term n = 4900	To assess neurodevelopmental outcomes from infancy to school age and determine predictive values of earlier developmental testing compared with school-age testing	24 months, 4–6 years (kindergarten) 24 months: MDI of BSF-R 4–6 years: TSRS Confounders: Maternal race, education, marital status, prenatal care, primary language, impoverished household, gender, fetal growth, plurality, delivery type, gestational age, and breastfeeding	- LPT >FT in aOR of worse TSRSs (aOR 1.52 (95%CI 1.06–2.18) -Positive predictive value of MDI <70 at 24 months and a TSRS <5% at 4–6 years was 10.4% **LPT continue to be delayed at kindergarten compared with FT. The predictive validity of having a TSRS in the bottom 5% given a MDI<70 at 24 months was poor. A child who tested within the normal range (>85) at 24 months had an excellent chance of testing in the normal range at kindergarten.**
Chan & Quigley, 2014. UK [49]	Cohort study Millenium Cohort Study (MCS) Born 2000–2001	VPT n = 69 MPT n = 67 LPT n = 360 ET n = 1258 Control group Term n = 4277	To investigate the effect of GA particularly in LPT and ET on school performance at 7 years	7 years -KS1 Confounders: Maternal age at delivery, maternal education, maternal socioeconomic status, marital status, multiple births, whether firstborn, smoking during pregnancy. Gender, age within school year.	Increased risk of poor performance: -VPT: aRR 1.78 (95%CI 1.24–2.54) -MPT: aRR 1.71 (95%CI 1.15–2.54) -LPT: aRR 1.36 (95%CI 1.09–1.68) -ET: aRR 1.07 (95%CI 0.94–1.23) **LPT birth negatively impacts academic outcomes at age 7 years as measured by KS1 school assessment.**
Sheehan et al., 2017. Canada [50]	Cohort study Born 2000–2011	ELBW n = 105 LPT n = 248 Control group FT n = 132	To examine the effect of preterm birth on planning skills in early and middle childhood using problem solving tasks with different cognitive workload demands	3, 6 and 9 years -Monkey Tree task (MTT) -DAS/DAS-II -Beery-Buktenica Developmental Test -B-HB -BRIEF-P Confounders: Gender, maternal education	- 3 years: LPT < FT in problem solving (MTT). - 6 to 9 years: LPT = FT in problem solving efficiency. Significant correlations between MTT measures and performance on other EF tasks. **MTT captured significant performance differences in planning skills between LPT and FT. Cognitive workload, as a function of problem complexity, affects planning skills in young LPT who show a subtle, but distinguishable, adverse neuropsychological outcome.**
Brumbaugh, et al., 2016. US [51]	Observational cohort study Born 2000–2006	LPT n = 52 Control group FT n = 74	To analyze the potential occurrence of altered brain development in LPT	6–13 years -PBS-30 -WISC-IV -WRAT -GPT-PANESS Confounders: No	-LPT < FT in processing speed, visuo-spatial perception and memory. -LPT > FT on behavioural difficulties from parental reports -LPT = FT in cognitive ability/academic achievement. -LPT = FT on intracranial volumes, but less total tissue and more cerebrospinal fluid in LPT. Tissue differences in the cerebrum are distributed across cortical and subcortical tissue. LPT had a relatively smaller thalamus than FT. Only FT demonstrated significant decreases in cortical tissue volume and thickness with age. **LPT demonstrated more difficulty in processing speed, visual-spatial perception, and memory. Together the behavioural, cognitive and brain structural findings suggest the potential insult of LPT birth on the developing brain given the differences persist at school age.**

ASQ: Ages and Stages Questionnaire; BRIEF-P: Behavior Rating Inventory of Executive Function-Preschool version; B-HB test: Baron-Hopkins Board test; BSF-R: Bayley Short Form-Research edition; CBCL: Child behavior Checklist; DAS: Differential Ability Scales; EF: executive function; ELBW: extremely low birth weight; EPT: early preterm; ET: early term; FSP: Foundation Stage Profile; FT: full-term; GA: gestational age; GPT: Grooved Pegboard Test; IQ: Intelligence Quotient; ITSEA: Infant Toddler Social Emotional Assessment;KS1: Key Stage 1; LPT: late preterm; MDI: Mental Developmental Index; MLPT: Moderate-late preterm; MPT: moderate preterm; MSD: Motor and Social Developmental Scale; MTT: Monkey Tree Task; NA: Not available; PBS-30: Pediatric Behavior Scale-30; P-CPT: Preschool Continuous Performance Test;: small for gestational age; PANESS: Physical and Neurological Examination for Soft Signs; PPVT: Peabody Picture Vocabulary Test; Pre-CTOPP: Preschool Comprehensive Test of Phonological and Print Processing; TEACh: Test of Everyday Attention for Children; TSRS: Total School Readiness Score; TVSC: Test of Visuospatial Construction; UTATE: Utrecht Tasks of Attention in Toddlers using Eye tracking; VPT: very preterm; WISC: Wechsler Intelligence Scale for Children; WPPSI: Wechsler Preschool and Primary Scale of Intelligence;: Wide Range Achievement Test.

**Table 2:** Summary of Critical Appraisal Skills Program (CASP) results.

Reference	Did the Study Address a Clearly Focused Issue?	Was the Cohort Recruited in An Acceptable Way?	Was the Exposure Accurately Measured to Minimize Bias?	Was the Outcome Accurately Measured to Minimize Bias?	Have the Authors Identified All Important Confounding Factors?	Have They Taken Account of the Confounding Factors in the Design and/or Analysis?	Was the Follow Up of Subjects Complete Enough?	Was the Follow Up of Subjects Long Enough?
Baron et al. [36]	Yes	Yes	Yes	Yes	No	Yes	No	No
Stene-Larsen et al. [37]	Yes	Yes	Yes	Yes	Yes	Yes	Yes	No
Brown et al. [38]	Yes	Yes	Yes	Yes	Yes	Yes	No	Yes
Brumbaugh et al. [39]	Yes	Yes	Yes	Yes	No	No	Yes	Yes
Shah et al. [40]	Yes	Yes	Yes	Yes	Yes	Yes	Yes	Yes
Sejer et al. [41]	Yes	Yes	Yes	Yes	Yes	Yes	Can’t tell	Yes
Hodel et al. [42]	Yes	Yes	Yes	Yes	No	No	Yes	Yes
Hornman et al. [43]	Yes	Yes	Yes	Yes	Yes	Yes	No	Yes
Quigley et al. [44]	Yes	Yes	Yes	Yes	Yes	Yes	No	Yes
Baron et al. [45]	Yes	Yes	Yes	Yes	No	No	Yes	Yes
Rider et al. [46]	Yes	Yes	Yes	Yes	No	No	Yes	Yes
Bogicevic et al. [47]	Yes	Yes	Yes	Yes	No	Yes	Yes	Yes
Woythaler et al. [48]	Yes	Yes	Yes	Yes	Yes	Yes	Yes	Yes
Chan &Quigley [49]	Yes	Yes	Yes	Yes	Yes	Yes	No	Yes
Sheehan et al. [50]	Yes	Yes	Yes	Yes	Yes	Yes	Yes	Yes
Brumbaugh et al. [51]	Yes	Yes	Yes	Yes	No	No	No	Yes
